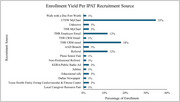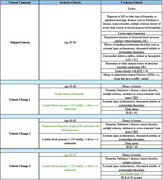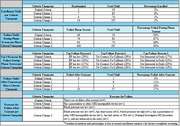# IPAT Study Enrollment via the Electronic Health Record “MyChart”

**DOI:** 10.1002/alz70860_106744

**Published:** 2025-12-23

**Authors:** Jordan Zaenglein, Kathleen Esselink, Hong Li, Margaret McGregor, Solymar Rivera‐Torres, Tristyn Hall, Rong Zhang

**Affiliations:** ^1^ Institute for Exercise and Environmental Medicine, Texas Health Presbyterian Hospital Dallas, Dallas, TX, USA; ^2^ University of Texas Southwestern Medical Center, Dallas, TX, USA

## Abstract

**Background:**

The Impact of Intensive Treatment of Systolic Blood Pressure (SBP) on Brain Perfusion, Amyloid, and Tau in Older Adults (IPAT) is an ongoing, NIH funded, randomized control trial (RCT) for prevention of Alzheimer's disease (AD) (R01AG076660). The primary outcome is to determine the effect of intensive lowering of high SBP on brain amyloid accumulation in older adults. The trial will enroll 180 participants and follow them for 2 years. Recruitment via Epic® MyChart® messaging, an electronic health record system (ERH) at the University of Texas Southwestern Medical Center (UTSW), has been one of the primary recruitment sources from 02/09/2023 to 12/09/2024 (Graph 1).

**Method:**

The UTSW‐EHR opt‐out system has 616,656 active patients who can be contacted for clinical research. To improve recruitment efficiency via the UTSW‐EHR, 4 screening criteria changes were implemented to determine which criteria had a high yield rate for study enrollment (Table 1).

**Result:**

Among other recruitment sources, MyChart yielded the highest study enrollment at 35% (Graph 1). The enrollment and phone screen failure rates remained stable despite the screening criteria changes (Table 2). For the original criteria, 2% were enrolled and 96% failed phone screening. For criteria change #1, 8% were enrolled and 87% failed phone screening. For criteria change #2, 5% were enrolled and 86% failed phone screening. For criteria change #3, 3% were enrolled and 90% failed phone screening. However, there was an increase in failure rate after in‐person consent as the total number of participants who passed an initial phone‐screen increased (Table 2).

**Conclusion:**

Electronic Health Record (MyChart) can be a valuable source for AD prevention trial recruitment with a careful selection of participant screening criteria.